# Constraining the carbonate system in soils via testing the internal consistency of pH, pCO_2_ and alkalinity measurements

**DOI:** 10.1186/s12932-020-00069-5

**Published:** 2020-03-30

**Authors:** Sima Bargrizan, Ronald J. Smernik, Luke M. Mosley

**Affiliations:** 1 The School of Agriculture, Food and Wine, The University of Adelaide, Waite Campus, Adelaide, SA 5064 Australia; 2 Acid Sulfate Soils Centre, School of Biological Sciences, The University of Adelaide, Adelaide, Australia

**Keywords:** Soil carbonate system, Internal consistency, Spectrophotometric method, Total alkalinity

## Abstract

Inorganic carbon exists in various dissolved, gaseous and solid phase forms in natural waters and soils. It is important to accurately measure and model these forms to understand system responses to global climate change. The carbonate system can, in theory, be fully constrained and modelled by measuring at least two of out of the following four parameters: partial pressure (pCO_2_), total alkalinity (TA), pH and dissolved inorganic carbon (DIC) but this has not been demonstrated in soils. In this study, this “internal consistency” of the soil carbonate system was examined by predicting pH of soil extracts from laboratory measurement of TA through alkalinity titration for solutions in which pCO_2_ was fixed through equilibrating the soil solution with air with a known pCO_2_. This predicted pH (pH_CO2_) was compared with pH measured on the same soil extracts using spectrophotometric and glass electrode methods (pH_spec and_ pH_elec_). Discrepancy between measured and calculated pH was within 0.00–0.1 pH unit for most samples. However, more deviation was observed for those sample with low alkalinity (≤ 0.5 meq L^−1^). This is likely attributable to an effect of dissolved organic matter, which can contribute alkalinity not considered in the thermodynamic carbonate model calculations; further research is required to resolve this problem. The effects of increasing soil pCO_2_ was modelled to illustrate how internally consistent models can be used to predict risks of pH declines and carbonate mineral dissolution in some soils.

## Introduction

Concentrations of atmospheric carbon dioxide (CO_2_) have increased by 40%, from 280 ppm in 1750 to 400 ppm in 2014 [1]. This increase has been caused by anthropogenic activities, especially burning of fossil fuels which is the main cause of global warming [2]. The soil inorganic carbon system is one of the largest sinks of atmospheric CO_2_ and the global C cycle [3], and is vulnerable to anthropogenic perturbations [4]. The partial pressure of CO_2_ (pCO_2_) in the atmosphere is in equilibrium with the soil surface but deeper soil layers may have higher pCO_2_ due to microbial respiration and -slow exchange with the atmosphere. Increasing soil pCO_2_ as a consequence of the increasing concentrations of atmospheric pCO_2_ [5, 6] has demonstrated the participation of soil inorganic carbon systems to global climate change.

Inorganic carbon can occur in different forms in soils including dissolved species (CO_2_, carbonic acid, bicarbonate, and carbonate ions), and solid mineral phases (e.g. calcium carbonate, dolomite). Calcium carbonate (CaCO_3_) can comprise a major part of some soil systems, particularly in arid and semi-arid areas [7]. Under increased soil pCO_2_, soil acidification occurs through carbonic acid formation followed by weak acid dissociation [8]. The weathering (dissolution) of CaCO_3_ (calcite and aragonite) in soils arises from either carbonic acid at pH > 6 or strong acids at lower pH. Dissolution of solid calcium carbonate at pH > 6.5 [9–11] provides a buffer (provided that it is not exhausted) via an increase in HCO_3_^−^ alkalinity against pH changes in soil [12] caused by acidification processes [13]. As well as pH the assessment of the degree of CaCO_3_ saturation is crucial for agricultural management due to its influence on chemical and physical soil characteristics such as cation exchange capacity (CEC), porosity, and hydraulic conductivity [14]. The outcome of anthropogenic climate change could be a decrease in CaCO_3_ mineral saturation status, resulting in dissolution [15–17], a decrease in the pH buffering capacity, and soil acidification.

To give increased confidence in predicting the effects of increased atmospheric pCO_2_ and risks of soil acidification, accurate characterization of the soil inorganic carbon system [18] is essential. This requires measurement of inorganic carbon system variables such total alkalinity (TA), pH, pCO_2_ and dissolved inorganic carbon (DIC) [19]. By measuring accurately at least two of these inorganic carbon system parameters it is possible to calculate the remaining parameters using knowledge of carbonate equilibrium constants [20]. If a third carbonate system parameter is measured this enables rigorous checking of the internal consistency of the equilibrium constants of the system and accuracy of measurements [21].

The internal consistency assists in checking if the same outcomes can be obtained through different independent carbonate system measurements [22]. The internal consistency of different sets of marine carbonate system measurements and equilibrium constants has previously been demonstrated [18, 23–26]. However, this internal consistency has not been demonstrated yet for the soil carbonate system, and this introduces major uncertainties in our ability to understand acidification risks and response to rising atmospheric CO_2_ levels. Highly precise analytical measurements of carbonate parameters are a prerequisite for evaluation of the internal consistency of this system [27–31]. This was one of the drivers for our recent development of spectrophotometric pH measurement methods for soil extracts [13, 32] which had previously been proven to provide high precision pH (< 0.01 pH units) in the marine chemistry field [33–38].

The objective of this study was to develop a model for evaluation of the consistency of thermodynamics of the soil carbonate system by calculation of a third parameter from two other parameters. Using a controlled laboratory experiment, we calculated pH of soil extracts equilibrated with a fixed pCO_2_ and measured total alkalinity (TA) and then compared the results with pH measured through spectrophotometric and glass electrode methods. This study is also unique in terms of the investigation of the internal consistency of the soil carbonate system through the incorporation of state-of-the-art spectrophotometric methods for pH measurement. A further aim of this study was to assess the accuracy of spectrophotometric soil pH measurements against conventional glass electrode pH measurements using the same approach. A modelling approach was then explored as a potential tool for prediction of increasing soil pCO_2_ and soil carbonate dissolution as a result of global climate change.

## Materials and methods

### Theory

#### Soil pH determination using acid–base equilibria of CO_2_

The pH and carbonate equilibria in the soil solution can in theory be determined using Henry’s Law constant for CO_2_ (K_H_), the first and second dissociation constants of carbonic acid (H_2_CO_3_*) (K_1_ and K_2_) resulting in bicarbonate and carbonate ions, respectively and the water self-dissociation equilibrium constant (K_w_) [39]:

1a $${\text{CO}}_{2} + {\text{H}}_{2} {\text{O }} \rightleftharpoons {\text{H}}_{2} {\text{CO}}_{3}^{ *} \quad {\text{K}}_{\text{H }} = \frac{{\left\{ {{\text{H}}_{2} {\text{CO}}_{3}^{ *} } \right\}}}{{f{\text{CO}}_{2} }} = 10^{ - 1.47}$$

1b $${\text{H}}_{2} {\text{CO}}_{3}^{ *} \rightleftharpoons {\text{H}}^{ + } + {\text{HCO}}_{3}^{ - } \quad {\text{K}}_{1 } = \frac{{\left\{ {{\text{H}}^{ + } } \right\}\left\{ {{\text{HCO}}_{3}^{ - } } \right\}}}{{\left\{ {{\text{H}}_{2} {\text{CO}}_{3}^{ *} } \right\}}} = 10^{ - 6.35}$$

1c $${\text{HCO}}_{3}^{ - } \rightleftharpoons {\text{H}}^{ + } + {\text{CO}}_{3}^{2 - } \quad {\text{K}}_{2 } = \frac{{\left\{ {{\text{H}}^{ + } } \right\}\left\{ {{\text{CO}}_{3}^{2 - } } \right\}}}{{\left\{ {{\text{HCO}}_{3}^{ - } } \right\}}} = 10^{ - 10.33}$$

1d $${\text{H}}_{2} {\text{O }} \rightleftharpoons {\text{H}}^{ + } + {\text{OH}}^{ - } \quad {\text{K}}_{\text{W }} = \left\{ {{\text{H}}^{ + } } \right\}\left\{ {{\text{OH}}^{ - } } \right\} = 10^{ - 14}$$

{} represents ion activities for the mass action equations with the equilibrium constants given valid at 25 °C and zero ionic strength (µ = 0). The fugacity (fCO_2_) may be approximated by the partial pressure of CO_2_(pCO_2_) in the air as the ideal behavior of CO_2_ was considered in our study.

The soil pH_CO2_ is determined using the following equation:

2 $${\text{Alkalinity }} = {\text{C}}_{\text{B }} - {\text{C}}_{\text{A }} = \frac{{K_{H} fCO_{2} }}{{\alpha_{0} }} \left( {a_{1} + 2a_{2} } \right) + \frac{{{\text{K}}_{\text{w}} }}{{\left[ {{\text{H}}^{ + } } \right]}} - \left[ {{\text{H}}^{ + } } \right]$$

This equation represents experimentally measured alkalinity since it corresponds to the concentration of strong acid required to titrate the solution to the endpoint of bicarbonate. pH can be determined using equation [2] provided that the amounts of acid or base added to the system and pCO_2_ are known. Equation (2), can be solved iteratively by the bisection method until the left-hand side (alkalinity) equals the right-hand side or via other numerical methods (see the supplementary information, Additional File 1, for the full derivation of the equation).

### Soil extract preparation

Nine soils collected from South Australia with a pH range of 6–8 (Table 1) and three replicates of each soil extract (1:1 w/v soil:water) were used in the study for pH measurements (refer to [32] for details).

**Table 1:** Soil physical properties and major ion concentrations in a 1:1 w/v soil:water extract

	Depth, cm	Sand silt clay, %			Major cations and anions						
					Cl^−^	NO_3_^−^	SO_4_^2−^	Ca^2+^	K^+^	Mg^2+^	Na^+^
					meq L^−1^						
Monarto 1*	0–10	84.6	7.10	8.30	0.65	0.44	0.13	2.62	0.46	0.56	0.69
Lock siliceous	0–10	95	0	5	0.38	2.35	0.09	3.59	0.78	0.44	0.35
Karoonda	0–10	97.4	0.2	2.40	0.24	0.24	0.11	0.39	0.25	0.21	0.20
Ngarkat	0–10	95.80	1.0	3.20	0.18	0.04	0.05	0.25	0.11	0.17	0.21
Lock Horizon B	0–10	97.50	2.50	0	0.20	0.34	0.14	1.40	0.11	0.54	0.60
Modra	0–10	65	5	30	3.36	5.70	0.31	5.72	1.34	1.39	1.34
Monarto 2*	0–10	93.6	1.1	3.8	0.31	0.24	0.19	0.50	0.39	0.30	0.25
Cowirra	0–10	41.50	18.80	39.70	4.57	0.02	35.6	25.46	1.14	14.36	9.17
Black point	10–20	72.70	9.20	18.10	2.21	0.28	0.37	2.23	0.27	0.55	2.46

*Monarto 1 and Monarto 2 were selected form two locations (Highland and Highway, respectively)

### Laboratory experimental set up

**Fig. 1:**
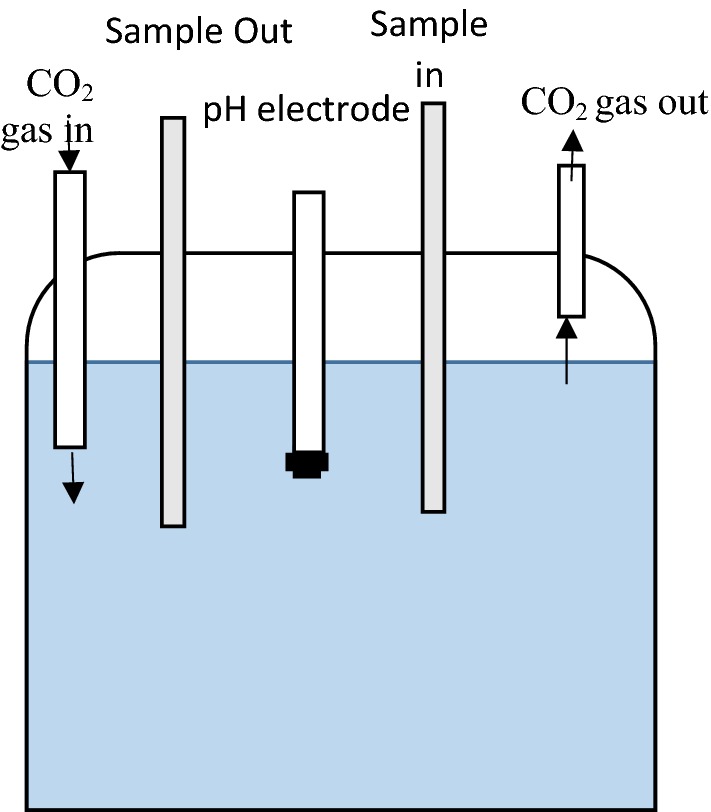
Diagram of soil carbonate equilibrium cell

A laboratory experiment was conducted in which ca. 25 mL of soil extract was introduced into a custom-made equilibration flask (Fig. 1) which was connected via tubing to a flow-through cell on a double-beam spectrophotometer (GBC UV/VIS 916).

The flask was placed on a temperature-controlled water bath adjusted to 25 °C. The temperature in the spectrophotometric cell holder was also kept constant at 25 °C using an installed water thermostat. A pH electrode (Orion SureFlow) was inserted into the flask that had been pre-calibrated with commercially manufactured (Australian Chemical Reagents) standard high ionic strength pH 7 and pH 4 buffers (m ≈ 0.1 mol L^−1^) at 25 °C.

The soil extracts were equilibrated with a fixed pCO_2_ via a gas tube connected to a pure air cylinder (BOC gases) inserted into the top of the equilibration cell (Fig. 1). The pCO_2_ in the gas stream was measured using a calibrated LICOR 840a infra-red gas analyser. The air was circulated through the soil solution using slow bubbling for approx. 30 min per sample until the spectra of solution and the electrode pH measurement were stable.

Then for spectrophotometric pH measurement, a sulfonephthalein indicator depending on the sample pH range (determined by the electrode) was selected and injected into the soil solution. The absorbance spectrum with dye was recorded for the circulating soil extract solution. The absorbance spectrum for the indicator was generated through subtraction of a baseline spectrum of soil solution without indicator dye (refer to [32] for details).

### Alkalinity measurement

After pH measurement, a measured volume of soil solution and indicator dye was transferred into a separate beaker for alkalinity measurement. Great attention was taken to avoid solution loss by extracting all the solution out of the flow-through cell and connecting tubes. The solution was stirred gently, and initial pH was recorded when a stable reading was obtained, and then titration was conducted using an autotitrator to deliver increments of 0.16 N H_2_SO_4_ and continued to the end point at pH ≤ 4.5. pH was measured using a glass electrode after each acid addition. Adequate titration points were recorded, ensuring high accuracy. A gran linear extrapolation function was utilized to determine alkalinity for low ionic strength samples [40].

### Laboratory analytical measurements

Stock solutions of bromocresol purple (BCP) and phenol red (PR) at a total concentration of 3 × 10^−3^ mol L^−1^ were used. The absorbance maxima (Abs) of acid and base forms of PR were read at 433 nm, 558 nm (λ_1_ and λ_2_) and BCP at 432 nm, 589 nm (λ_1_ and λ_2_), respectively, using the spectrophotometer Cintral™ software and used for R (= λ2Abs./λ1Abs) calculation (see [32]). The value for molar absorbance ratios (e_1_-e_3_) and pK_2_ of indicators used in this study (PR and BCP) are those of [36].

The ionic strength (µ) of each soil extract was determined via electrical conductivity (EC, mS cm^−1^) measurement using a calibrated conductivity electrode (TPS Glass K = 1.0 Cond Sensor) using the equation µ = EC × 0.0127 [32, 41, 42].

The concentration of dissolved organic carbon (DOC) in filtered soil solutions was also estimated using a spectrophotometer at an absorbance of 250 nm [43] using the regression equation [DOC] = 33.99 A_250_ + 8.16 [43–45].

Concentrations of major cations (Ca^2+^, Mg^2+^, Na^+^, K^+^) were measured by inductively coupled plasma optical emission spectroscopy (ICPOES) [46] and concentrations of anions (NO_3_^−^, SO_4_^2−^, Cl^−^) were determined by ion chromatography using a Dionex ICS-2500 system [46] (Table 1).

### Geochemical modelling calculations

To assess the internal consistency of the soil carbonate system, we compared the soil solution pH (n = 27, pH range of appx. 6–8) calculated from pCO_2_ (pH_CO2_), and alkalinity measurements using Eq. (2), to pH measured using both spectrophotometric (pH_spec_) and glass electrode (pH_elec_) methods. Carbonate system calculations were based on equilibrium constants reported by [39] at zero ionic strength (µ = 0) and 25 °C with corrections for variable ionic strength made using the Davies equation.

The geochemical speciation program PHREEQC [47] was used to calculate carbonate mineral (calcite, aragonite, dolomite) saturation states from the fixed pCO_2_ and measured alkalinity, measured major ions and also at a range of elevated pCO_2_ (to assess the effect of climate change) values.

## Results and discussion

### Measurement and internal consistency of the soil carbonate system

The pH values calculated from pCO_2_ and alkalinity (pH_CO2_) and pH obtained using electrode and spectrophotometric methods (pH_elec_ and pH_spec_) are shown in Table 2. An average precision of ca. 0.03 pH units was obtained for three replicate (pH_CO2_) measurements, which was similar to the recorded precision of measured pH_spec_ and pH_elec_ values (0.05 pH units) (Table 2). Hence the potential for spectrophotometric pH measurements to provide a higher accuracy for determining the carbonate system parameters (compared to a conventional glass electrode measurement) was not proven in this experiment. This may reflect our very careful pH electrode measurement protocols (e.g., temperature control, electrodes with free-flowing junctions designed for soil). Spectrophotometrically measured pH along with another carbonate system parameter has been the most common and accurate approach to calculate oceanic pCO_2_ [23, 26]. This might be particularly important in saline soils where there are difficulties in calibrating glass electrodes to enable accurate measurement.

**Table 2:** Mean and standard deviation (SD) of calculated pH (pH_CO2_), measured pH_spec_ and pH_elec_ in different soils

Soil	pH_CO2_ (SD)	pH_spec_ (SD)	pH_elec_ (SD)
Lock Siliceous	7.98 (0.01)	8.00 (0.09)	7.91 (0.03)
Ngarkat	7.08 (0.01)	7.03 (0.04)	6.79 (0.03)
Monarto 1	8.06 (0.01)	8.06 (0.03)	8.11 (0.04)
Modra	7.60 (0.04)	7.67 (0.04)	7.63 (0.02)
Lock Horizon	8.17 (0.03)	8.12 (0.04)	8.10 (0.04)
Karoonda	7.05 (0.06)	6.47 (0.08)	6.24 (0.14)
Monarto 2	7.17 (0.12)	6.75 (0.06)	6.63 (0.06)
Cowirra	7.79 (0.01)	7.74 (0.05)	7.72 (0.02)
Black point	7.94 (0.01)	8.04 (0.03)	8.04 (0.01)
Average SD	0.03	0.05	0.05

**Fig. 2:**
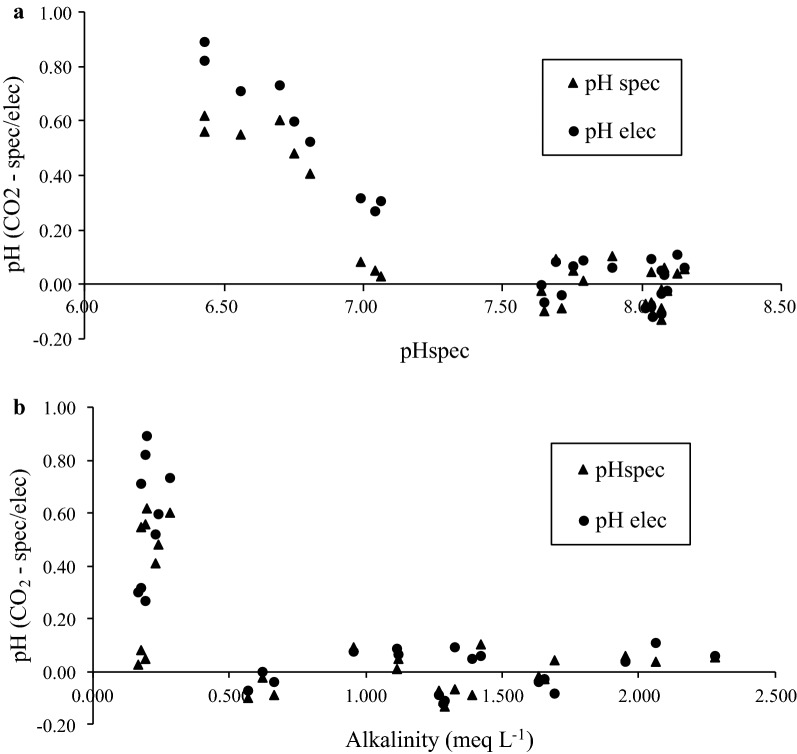
Difference between pH calculated using carbonate systems and spectrophotometric and electrode pH measurements for different soils against spectrophotometric pH values (**a**). Difference between calculated and measured pH values as a function of total alkalinity (**b**)

A plot of the residual of pH_CO2_ minus pH_elec_, pH_spec_ (Fig. 2a), shows that calculated pH (pH_CO2_) was in general higher than the measured pH values (pH_elec_ and pH_spec_). There was a good agreement between measured and calculated pH for soil extracts with pH > 7 (Table 2, Fig. 2a) with an average difference of approximately 0.1 pH units. These results show that the soil carbonate system model using the constants of [39] was internally consistent with measurements in pH > 7 extracts. While the internal consistency of the seawater CO_2_ system has been previously demonstrated [18, 23, 26, 27, 31], our measurements show it is possible to demonstrate this in soil solutions. This is important as it demonstrates, for the first time to our knowledge, that carbonate system equilibria can be accurately modelled in soils.

However, there was a larger deviation of 0.3–0.8 pH units for those samples with pH ≤ 7 (Table 2, Fig. 2a), which mainly corresponded to soil extracts with low alkalinity of < 0.5 meq L^−1^ (Fig. 2b). Inconsistencies in the pH-DIC-pCO_2_ relationship have been previously explained with regards to the difference between TA through the carbonate model and measured TA concentrations [26]. Measured TA constitutes the contribution of both organic bases [26, 30, 31, 48] and carbonate species. Conversely, calculation of TA via the thermodynamic carbonate model used in this study does not include the contribution of organic bases as they cannot be easily measured. However, in an attempt to determine the source of total alkalinity surplus relative to calculated carbonate, dissolved organic carbon (DOC) for all samples was estimated via spectrophotometric measurements in the UV-range [43] (Table 3). There was little difference DOC among the samples, with all extracts containing approximately 70 mg L^−1^ DOC, except Lock B (33.5 mg L^−1^).

**Table 3:** The mean value of alkalinity titration (TA_tit_) with standard deviation (SD) in brackets and estimated dissolved organic carbon (DOC) in different soils

Soil	TA_tit_ (SD), meq L^−1^	Estimated DOC, mg L^−1^
Lock siliceous	1.38 (0.05)	74.69
Monarto 1	1.66 (0.03)	78.14
Ngarkat	0.18 (0.01)	62.08
Modra	0.62 (0.05)	68.88
Lock B	2.9 (0.17)	33.50
Karoonda	0.19 (0.01)	70.91
Monarto 2	0.25 (0.03)	73.71
Cowirra	1.06 (0.09)	76.64
Black point	1.28 (0.01)	75.24

**Fig. 3:**
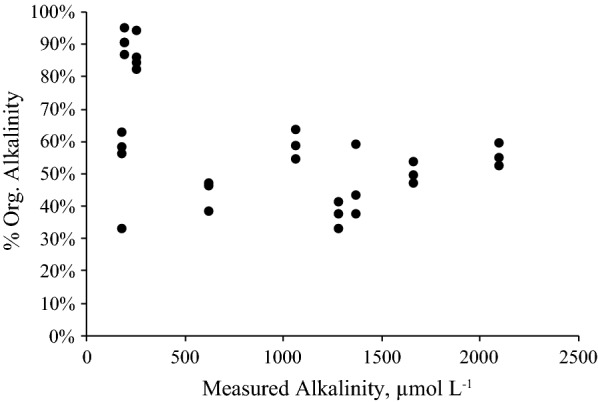
Organic alkalinity (%) against measured total alkalinity (µmol L^−1^)

For those samples with low alkalinity ≤ 0.5 meq L^−1^ the discrepancy between total (Gran, measured alkalinity) and carbonate alkalinity (calculated TA using the model) seems likely to have been caused by the uptake of protons by organic bases (Table 2). To further assess this, the organic alkalinity of soil solutions was estimated from the difference between measured total alkalinity and carbonate alkalinity calculated using the thermodynamic carbonate model (Eq. 2) with the measured spectrophotometric pH and experimentally fixed pCO_2_ as inputs. The  % of organic alkalinity versus total alkalinity shown in Fig. 3 suggests that the low alkalinity soils in general have a much higher proportion of organic alkalinity. Hence it may be preferable to not use alkalinity as a measured parameter for carbonate system calculations in some soils with low alkalinity. Measuring another parameter of the soil carbonate system such as pCO_2_ or dissolved inorganic carbon (DIC) could be preferable in these soils. Spectrophotometric carbonate ion measurements have recently been developed [49] and may enable precise values that can be used for internal consistency calculations in soils.

### Calculation of carbonate mineral saturation states

Calcite, aragonite and dolomite saturation states (mg L^−1^) of soil solutions were calculated from non-fixed pCO_2_ (pH_spec_ and TA) and fixed pCO_2_ (pCO_2_ and TA) using the geochemical speciation program PHREEQC. There was variability in carbonate mineral saturation states with some over-saturated (SI > 0 at pH_PHREEQC_ > 8, suggesting mineral calcite could precipitate from solution) and some under-saturated (SI < 0 at pH_PHREEQC_ < 8, suggesting calcite dissolution) (Table 4).

**Table 4:** Calculated pH (pH_PHREEQC_) and calcite, aragonite and dolomite saturation status using the geochemical speciation program PHREEQC

Soil	pH _PHREEQC_	SI-Calcite, Aragonite and Dolomite (pH_spec_ and TA) (mg L^−1^)	SI-Calcite, Aragonite and Dolomite (pCO_2_ and TA) (mg L^−1^)
Lock Siliceous	8.27	0.28, 0.14, − 0.20	0.54, 0.39, 0.30
Monarto 1	8.36	0.34, 0.19, 0.14	0.62, 0.48, 0.71
Ngarkat	7.41	− 2.5, − 2.7, − 5.20	− 2.20, − 2.3, − 4.4
Modra	7.89	− 0.28, − 0.42, − 1.04	− 0.06, − 0.21, − 0.61
Lock Horizon	8.47	0.25, 0.10, 0.22	0.58, 0.43, 0.88
Karoonda	7.38	− 2.9,− 3.14, − 6.12	− 2.09, − 2.2, − 4.3
Monarto 2	7.51	− 2.4923, − 2.6361, − 5.0855	− 1.7, − 1.8, − 3.5
Cowirra	8.04	0.35, − 0.02, − 0.23	0.64, 0.17, 0.16

### Simulating increasing pCO_2_ in soil solutions

**Fig. 4:**
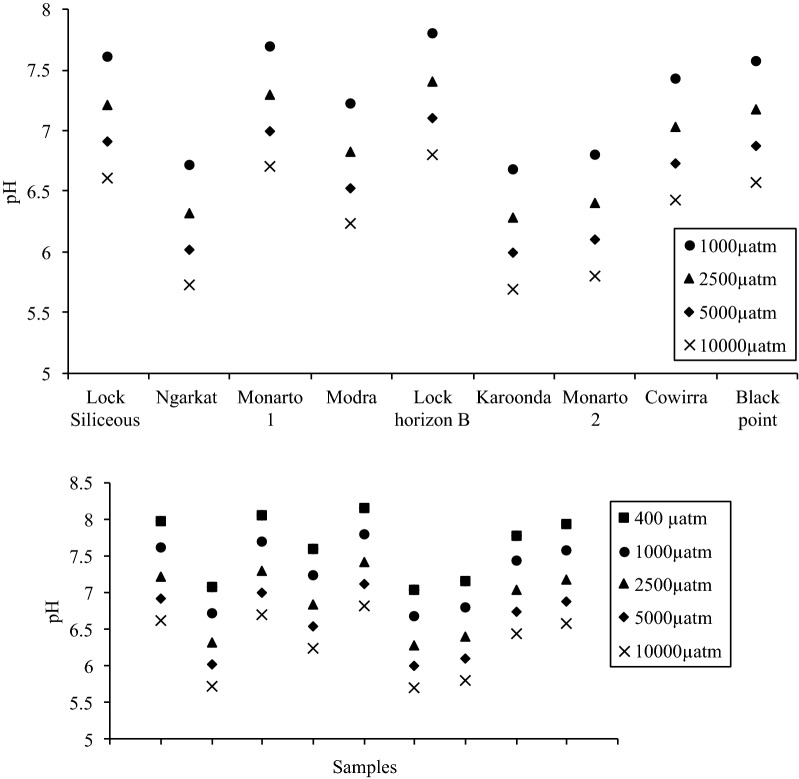
The mean pH calculated for the different pCO_2_ (1000, 2500, 5000, 10,000 µatm pCO_2_) concentrations using the carbonate system model for the 9 soil samples

**Fig. 5:**
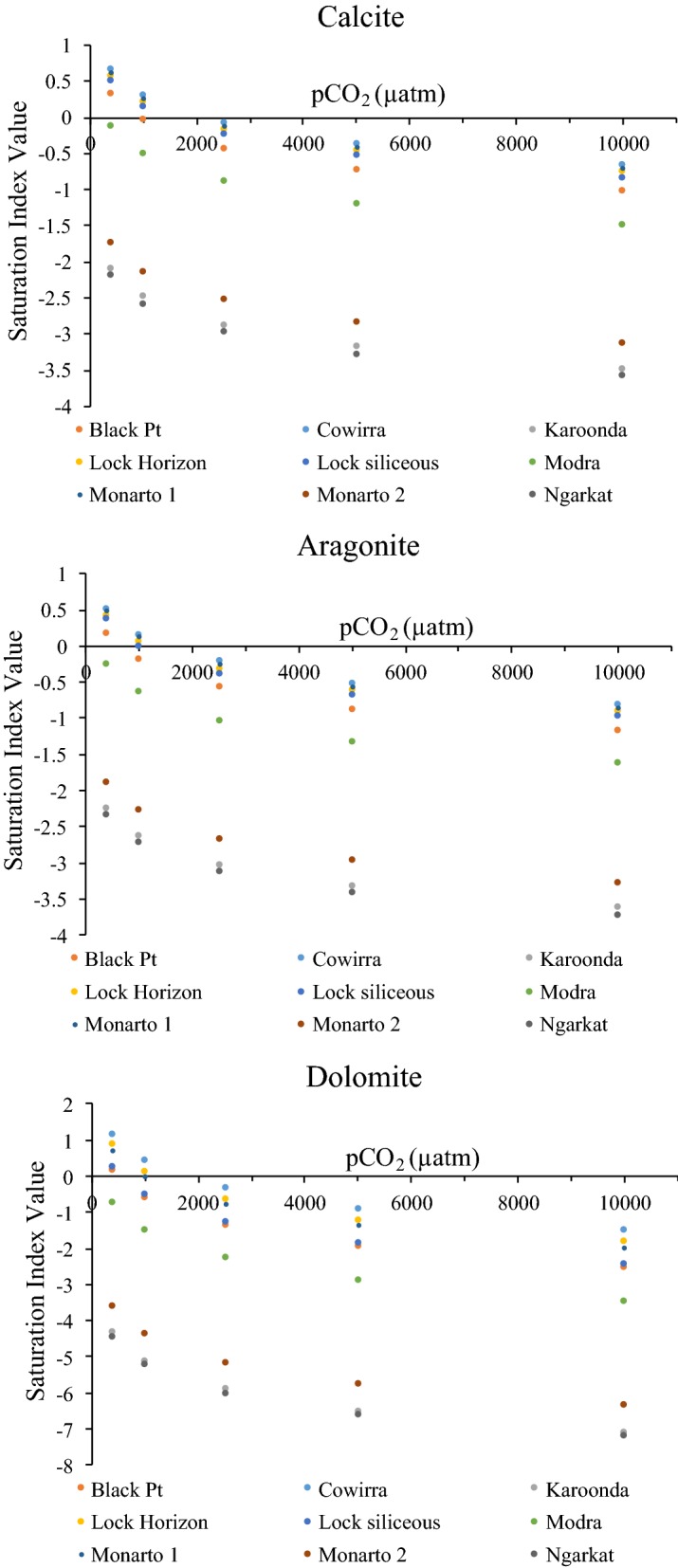
Modelled relationship between calcite, aragonite and dolomite saturation state and soil pCO_2_

As noted above soil pCO_2_ is one of the most important variables governing soil solution pH [50] and this is also influenced by atmospheric pCO_2_ changes. To indicate the potential application of an internally consistent carbonate system model to assess climate and/or biogeochemical process change effects, we modelled the influence of four elevated soil pCO_2_ scenarios (1000, 2500, 5000, and 10,000 µatm). Soil pH decreased from 0.4 to 1 pH units as a consequence of increased pCO_2_ levels from 1000 to 10,000 µatm respectively (Fig. 4). The effect of elevated pCO_2_ on calcite/aragonite (CaCO_3_) and dolomite (CaMg(CO_3_)_2_) saturation status (calculated using PHREEQC from pH, alkalinity and major ion concentrations) of these soils is shown in Fig. 5. The soils that are initially supersaturated relative to carbonate minerals at current atmospheric pCO_2_ levels (≈ 400 µatm) transition to undersaturated in the 500–1000 µatm pCO_2_ range. This is because carbonate ion concentrations have declined due to the lowering of pH (Fig. 4). In contrast the soils that are undersaturated at current atmospheric pCO_2_ levels (Karoonda, Ngarkat, Modra, Monarto 2) remain undersaturated as expected under elevated pCO_2_. This suggests that carbonate minerals in some surface soils could be highly vulnerable to dissolution due to global climate change, which could result in large pH changes once this buffer is exhausted. It is also important to note that our measurement set-up and models assumed an open system with fixed pCO_2_. Nevertheless, the internal consistency demonstrated should also apply to a closed system where pCO_2_ can vary while DIC is fixed.

The soil carbonate system measurement and modelling conducted in this study is an important first step for enabling a better understanding of related soil geochemical processes, in particular risks of inorganic carbonate dissolution due to global climate change. However, it would be important to see if internal consistency can also be demonstrated in situ. For this purpose, field experiments are now suggested accompanied by both spectrophotometric and electrode soil pH measurement methods. The influence of soil respiration, which affects both pCO_2_ and pH, also needs to be considered in the design of field measurements. Widespread global measurement of soil pH and calcium carbonate states using these methods would appear beneficial to assess the soil system response to climate change.

## Conclusions

In this study, the internal consistency of the soil carbonate system was assessed using experimental measurements and a thermodynamic equilibrium carbonate system model. The pCO_2_ was fixed in the experiment by equilibrating the soil solution with air with a known pCO_2_. Discrepancy of calculated pH from measured pH using spectrophotometric and glass electrode methods was within 0.00–0.1 pH units when alkalinity was > 0.5 meq L^−1^. This implies accurate prediction of pH from other carbonate system parameters is feasible using inorganic carbon system equilibrium calculations. However, contribution of organic bases appeared to result in errors in the calculated pH for samples with low alkalinity < 0.5 meq L^−1^. Nevertheless, this appears to be the first time that the internal consistency of soil carbonate system has been demonstrated. This enables a better understanding of soil responses to global climate change. Further development and application of methods for low alkalinity or organic-rich soils, and in situ measurement, is recommended.

## Supplementary information

**Additional file 1. **Includes theory and derivation of equations that underpin soil pH determinations using acid-base equilibria of CO_2_.

## Data Availability

All results of this article are available in the manuscript and figures, and also in the Ph.D. thesis of Sima Bargrizan.

## Abbreviations

TA: Total alkalinity
DIC: Dissolved inorganic carbon
pH_CO2_: Calculate pH using the carbonate model
pH_spec and_ pH_elec_: Measured pH using spectrophotometric and glass electrode methods
DOC: Dissolved organic carbon
pH_PHREEQC_: pH calculated by PHREEQC program
EC: Electrical conductivity
TA_tit_: Alkalinity titration
SD: Standard deviation
BCP: Bromocresol purple
PR: Phenol red

## References

[1] MacFarling Meure C, Etheridge D, Trudinger C, Steele P, Langenfelds R, van Ommen T, Smith A, Elkins J (2006). Law Dome CO_2_, CH_4_ and N2O ice core records extended to 2000 years BP. Geophys Res Lett.

[2] Pierre M (2014). Dynamics of the carbonate system and air-sea CO_2_ fluxes in western European shelf waters: a multi-scale approach.

[3] Lal R (2001). World cropland soils as a source or sink for atmospheric carbon. Adv Agron.

[4] Lal R, Kimble JM, Lal R, Kimble JM, Eswaran H, Stewart BA (2000). Pedogenic carbonates and the global carbon cycle. Global climate change and pedogenic carbonates.

[5] Andrews JA, Schlesinger WH (2001). Soil CO_2_ dynamics, acidification, and chemical weathering in a temperate forest with experimental CO_2_ enrichment. Global Biogeochem Cycles.

[6] King JS, Pregitzer KS, Zak DR, Sober J, Isebrands JG, Dickson RE, Hendrey GR, Karnsoky DF (2001). Fine-root biomass and fluxes of carbon in young stands of paper birch and trembling aspen as affected by elevated atmospheric CO_2_ and tropospheric O_3_. Oecologia.

[7] Strawn D, Bohn HL, O'Connor G (2015). Soil chemistry.

[8] Simunek J, Suarez DL (1993). Modeling of carbon dioxide transport and production in soil. 1. Model development. Water Resour.

[9] Kilham P (1982). Acid precipitation: its role in the alkalization of a lake in Michigan. Limnol Oceanogr.

[10] Perrin AS, Probst A, Probst JL (2008). Impact of nitrogenous fertilizers on carbonate dissolution in small agricultural catchments: implications for weathering CO_2_ uptake at regional and global scales. Geochim Cosmochim Acta.

[11] Raymond PA, Hamilton SK (2018). Anthropogenic influences on riverine fluxes of dissolved inorganic carbon to the oceans. Limnol Oceanogr Lett.

[12] Reardon EJ, Allison GB, Fritz P (1979). Seasonal chemical and isotopic variations of soil CO_2_ at Trout Creek. Ontario J Hydrol.

[13] Bargrizan S, Smernik RJ, Mosley LM (2018). The application of a spectrophotometric method to determine pH in acidic (pH < 5) soils. Talanta.

[14] Peverill KI, Sparrow LA, Reuter DJ (2001). Soil Analysis an Interpretation Manual.

[15] Berner RA (1997). The rise of plants and their effect on weathering and atmospheric CO_2_. Science.

[16] Bormann BT, Wang D, Bormann FH, Benoit G, April R, Snyder R (1998). Rapid plant-induced weathering in an aggrading experimental ecosystem. Biogeochemistry.

[17] Berg A, Banwart SA (2000). Carbon dioxide mediated dissolution of Ca-feldspar: implications for silicate weathering. Chem Geol.

[18] Wanninkhof R, Lewis E, Feely RA, Millero FJ (1999). The optimal carbonate dissociation constants for determining surface water pCO_2_ from alkalinity and total inorganic carbon. Mar Chem.

[19] Karberg NJ, Pregitzer KS, King JS, Friend AL, Wood JR (2005). Soil carbon dioxide partial pressure and dissolved inorganic carbonate chemistry under elevated carbon dioxide and ozone. Oecologia.

[20] Dickson A.G, Sabine CL, Christian JR (2007) Guide to Best Practices for Ocean CO_2_ measurements. PICES Special Publication 3, No 8. IOCCP Report.

[21] Marion GM, Millero FJ, Camões MF, Spitzer P, Feiste R, Chen CTA (2011). pH of Seawater. Mar Chem.

[22] Reimer JJ, Cai WJ, Xue L, Vargas R, Noakese S, Hu X, Signorini SR, Mathis JT, Feely RA, Sutton AJ, Sabine C, Musielewicz S, Chen B, Wanninkhof R (2017). Time series pCO_2_ at a coastal mooring: internal consistency, seasonal cycles, and interannual variability. Cont Shelf Res.

[23] Clayton TD, Byrne RH, Breland JA, Feely RA, Millero FJ, Campbell DM, Murphy PP, Lamb MF (1995). The role of pH measurements in modern oceanic CO_2_-system characterizations: precision and thermodynamic consistency. Deep-Sea Resh II..

[24] Zhang H, Byrne RH (1996). Spectrophotometric pH measurements of surface seawater at in situ conditions: absorbance and protonation behavior of thymol blue. Mar Chem.

[25] Lueker TJ, Dickson AG, Keeling CD (2000). Ocean pCO_2_ calculated from dissolved inorganic carbon, alkalinity, and equations for K_1_ and K_2_: validation based on laboratory measurements of CO_2_ in gas and seawater at equilibrium. Mar Chem.

[26] Patsavas MC, Byrne RH, Yang B, Easley RA, Wanninkhof R, Liu X (2015). Procedures for direct spectrophotometric determination of carbonate ion concentrations: measurements in the US Gulf of Mexico and East Coast Waters. Mar Chem.

[27] Millero FJ, Byrne RH, Feely RWR, Clayton T, Murphy F, Marilyn F, Lamb D (1993). The internal consistency of CO_2_ measurements in the equatorial Pacific. Mar Chem.

[28] Lee C, Wakeham SG, Hedges JI (2000). Composition and flux of particulate amino acids and chloropigments in equatorial Pacific seawater and sediments. Deep-Sea Res. I: Oceanogr Res Pap.

[29] Koeve W, Oschlies A (2012). Potential impact of DOC accumulation on fCO_2_ and carbonate ion computations in ocean acidification experiments. Biogeosci.

[30] Hoppe CJM, Langer G, Rokitta SD, Wolf-Gladrow DA, Rost B (2012). Implications of observed inconsistencies in carbonate chemistry measurements for ocean acidification studies. Biogeosci.

[31] Salt LA, Thomas H, Bozec Y, Alberto V, Borges AV, de Baar HJW (2016). The internal consistency of the North Sea carbonate system. J Mar Sys.

[32] Bargrizan S, Smernik RJ, Mosley LM (2017). Development of a spectrophotometric method for determining pH of soil extracts and comparison with glass electrode measurements. Soil Sci Soc Am.

[33] Robert-Baldo GL, Morris MJ, Byrne RH (1985). Spectrophotometric determination of seawater pH using phenol red. Analyt Chem.

[34] Byrne RH, Robert-Baldo G, Thompson SW, Chen CTA (1988). Seawater pH measurements: an at-sea comparison of spectrometric and potentiometric methods. Deep-Sea Res. Part.

[35] Clayton TD, Byrne RH (1993). Spectrophotometric seawater pH measurements: total hydrogen ion concentration scale calibration of m-cresol purple and at-sea results. Deep-Sea Res. Part I.

[36] Yao W, Byrne RH (2001). Spectrophotometric determination of freshwater pH using bromocresol purple and phenol red. Environ Sci Technol.

[37] Ohline SM, Reid MR, Husheer SL, Currie KI, Hunter KA (2007). Spectrophotometric determination of pH in seawater off Taiaroa Head, Otago, New Zealand: full-spectrum modelling and prediction of pCO_2_ levels. Marine Chem.

[38] Lai CZ, DeGrandpre MD, Wasser BD, Brandon TA, Clucas DS, Jaqueth EJ, Benson ZD, Beatty CM, Spaulding RS (2016). Spectrophotometric measurement of freshwater pH with purified meta-cresol purple and phenol red. Limnol Oceanogr Methods.

[39] Stumm W, Morgan J (1996). Aquatic chemistry: Chemical equilibria and rates in natural waters.

[40] Rounds. Alkalinity and acid neutralizing capacity. US Geological Survey TWRI Book

[41] Griffin B, Jurinak JJ (1973). Estimation of activity coefficients from the electrical conductivity of natural aquatic systems and soil extracts. Soil Sci.

[42] Gillman GP, Bell LC (1978). Soil solution studies on weathered soils from tropical North Queensland. Aust J Soil Res.

[43] Baldwin DS (1999). Dissolved organic matter and phosphorus leached from fresh and `terrestrially’ aged river red gum leaves: implications for assessing river-floodplain interactions. Freshwater Biol.

[44] O’Connell M, Baldwin DS, Robertson AI, Rees G (2000). Release and bioavailability of dissolved organic matter from floodplain litter: influence of origin and oxygen levels. Freshwater Biol.

[45] Whitworth KL, Baldwin DS, Kerr JL (2014). The effect of temperature on leaching and subsequent decomposition of dissolved carbon from inundated floodplain litter: implications for the generation of hypoxic blackwater in lowland floodplain rivers. Chem Ecol.

[46] APHA (2005). Standard Methods for the Examination of Water and Wastewater.

[47] der Helm AWC, Rietveld LC (2002). Modelling of drinking water treatment processes within the Stimela environment. Water Sci Technol.

[48] Kim HC, Lee K (2009). Significant contribution of dissolved organic matter to seawater alkalinity. Geophys Res Lett.

[49] Easley RA, Patsavas MC, Byrne RH, Liu X, Feely RA (2013). Mathis JT (2013) Spectrophotometric measurement of calcium carbonate saturation states in seawater. Environ Sci Technol.

[50] Robbins CW (1986). Carbon dioxide partial pressure in lysimeter soils. Agron.

