# Correction to: Constraining the carbonate system in soils via testing the internal consistency of pH, pCO_2_ and alkalinity measurements

**DOI:** 10.1186/s12932-020-00071-x

**Published:** 2020-04-22

**Authors:** Sima Bargrizan, Ronald J. Smernik, Luke M. Mosley

**Affiliations:** 1 The School of Agriculture, Food and Wine, The University of Adelaide, Waite Campus, Adelaide, SA 5064 Australia; 2 Acid Sulfate Soils Centre, School of Biological Sciences, The University of Adelaide, Adelaide, Australia

## Correction to: Geochem Trans (2020) 21:410.1186/s12932-020-00069-5

The original version of this article unfortunately contained a mistake. The presentation of Fig. 4 was incorrect. That is, in Fig. 4, the bottom graph in the figure should be removed.

The correct version of Fig. 4 is given below.

**Fig. 4:**
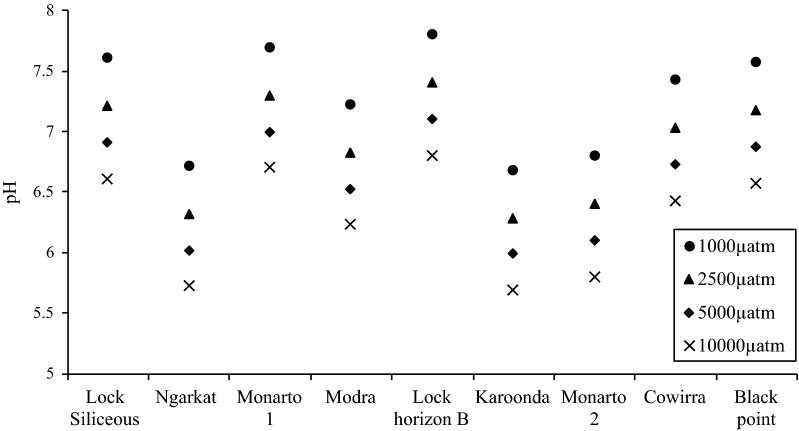
The mean pH calculated for the different pCO_2_ (400, 1000, 2500, 5000, 10,000 µatm pCO_2_) concentrations using the carbonate system model for the 9 soil samples

The original article has been corrected [1].
