# Supplementary material for: Constraining the carbonate system in soils via testing the internal consistency of pH, pCO2 and alkalinity measurements
Source: Geochem Trans. 2020 Mar 30;21:4. doi: 10.1186/s12932-020-00069-5 (PMC7106811; doi:10.1186/s12932-020-00069-5)
Supplement: Supplementary file 1 — Additional file 1. Includes theory and derivation of equations that underpin soil pH determinations using acid-base equilibria of CO2. [file 12932_2020_69_MOESM1_ESM.docx]

**Supplementary information for: Constraining the carbonate system in soils via testing the internal consistency of pH, pCO_2_ and alkalinity measurements**

**Sima Bargrizan^a^, Ronald J. Smernik^a^, Luke M. Mosley^b^,**

^a^The School of Agriculture, Food and Wine, The University of Adelaide, South Australia

^b^Acid Sulfate Soils Centre, School of Biological Sciences, The University of Adelaide, South Australia

**Appendix 1: Theory and derivation of equations that underpin soil pH determination using acid-base equilibria of CO_2_**

The pH and carbonate equilibria in the soil solution can in theory be determined using Henry’s Law constant for CO_2_ (K_H_), the first and second dissociation constants of carbonic acid (H_2_CO_3_*) (K_1_ and K_2_) resulting in bicarbonate and carbonate ions, respectively and the water self-dissociation equilibrium constant (K_w_)[1]:

$\mathrm{CO}_{2}+ H_{2}O \rightleftharpoons H_{2}\mathrm{CO}_{3}^{*}$ $K_{H}= \frac{\left\{ H_{2}\mathrm{CO}_{3}^{*} \right\}}{{f\mathrm{CO}}_{2}}= {10}^{-1.47}$ (1a)

$H_{2}\mathrm{CO}_{3}^{*} \rightleftharpoons H^{+}+ \mathrm{HCO}_{3}^{-}$ $K_{1}= \frac{\left\{ H^{+} \right\}\left\{ \mathrm{HCO}_{3}^{-} \right\}}{\left\{ H_{2}\mathrm{CO}_{3}^{*} \right\}}= {10}^{-6.35}$ (1b)

$\mathrm{HCO}_{3}^{-} \rightleftharpoons H^{+}+ \mathrm{CO}_{3}^{2-}$ $K_{2}= \frac{\left\{ H^{+} \right\}\left\{ \mathrm{CO}_{3}^{2-} \right\}}{\left\{ \mathrm{HCO}_{3}^{-} \right\}}= {10}^{-10.33}$ (1c)

$H_{2}O \rightleftharpoons H^{+}+\mathrm{OH}^{-}$ $K_{W}= \left\{ H^{+} \right\}\left\{ \mathrm{OH}^{-} \right\}= {10}^{-14}$ (1d)

The net negative charge arising from the dissociation of dissolved carbonic acid and other weak acids such as water (i.e. HCO_3_^-^, CO_3_^2-^, OH^-^) has to balance exactly the net positive charge from the strongly basic mineral cations (Na^+^, K^+^, Ca^2+^, Mg^2+^) and can be expressed as a charge balance or electroneutrality equation:

$C_{B}+\left[ H^{+} \right]= \left[ \mathrm{HCO}_{3}^{-} \right]+ 2\left[ \mathrm{CO}_{3}^{2-} \right]+ \left[ \mathrm{OH}^{-} \right]+ C_{A}$ (2a)

Where C_B_ and C_A_ are the amounts of base and acid that are present in the system, respectively. Because it is usually impractical to measure the amounts of acid and base that have been added, a new quantity, alkalinity, is defined representing the acid neutralizing capacity of the system. In carbonate-alkalinity dominated systems, the individual ions contributing to alkalinity may be expressed as:

Alkalinity = $C_{B}- C_{A}=\left[ \mathrm{HCO}_{3}^{-} \right]+2\left[ \mathrm{CO}_{3}^{2-} \right]+ \left[ \mathrm{OH}^{-} \right]-\left[ H^{+} \right]$ (2b)

In actual soil-water systems, other anions (e.g. sulfate) and organic bases may also provide alkalinity [2].

In an open system in close contact with the atmosphere (e.g. surface soil), the carbonic acid activity in a soil solution is governed by the partial pressure of CO_2_(g) (pCO_2_) in the gas phase. Therefore, the concentration of carbonate ions based on mass balance can be defined as [1]:

$\left[ H_{2}\mathrm{CO}_{3}^{*} \right]= C_{T}\alpha_{0}= K_{H}{f\mathrm{CO}}_{2}$ (3a)

Therefore $C_{T}= \frac{K_{H}{fCO}_{2}}{\alpha_{0}}$

$\left[ \mathrm{HCO}_{3}^{-} \right]= C_{T}\alpha_{1}$ (3b)

$\left[ \mathrm{CO}_{3}^{2-} \right]= C_{T}\alpha_{2}$ (3c)

Where, according to carbonate dissociation constants:

$\alpha_{0= \frac{1}{1+\frac{K_{1}^{'}}{\left\{ H^{+} \right\}}+ \frac{K_{1}^{'}K_{2}^{'}}{\left\{ H^{+} \right\}^{2}}}}$ (4a)

$\alpha_{1= \frac{1}{\frac{\left\{ H^{+} \right\}}{K_{1}^{'}}+1+\frac{K_{2}^{'}}{\left\{ H^{+} \right\}}}}$ (4b)

$\alpha_{2= \frac{1}{\frac{\left\{ H^{+} \right\}^{2}}{K_{1}^{'}K_{2}^{'}}+\frac{\left\{ H^{+} \right\}}{K_{2}^{'}}+1}}$ (4c)

K´_H_, K´_α1_ and K´_α2_ can be recalculated considering the effect of ionic strength on activity coefficients using the Davies equation [39]. (Refer to equation 1 for value of K_H_, K_1_ and K_2_ at zero ionic strength).

Substitution into the charge balance equation [2a] and combining equation [3a] for C_T_, [3b] and [3c] results in [5]:

Alkalinity = $C_{B}- C_{A}=\frac{K_{H}{fCO}_{2}}{\alpha_{0}} \left( a_{1}+2a_{2} \right)+ \frac{K_{w}}{\left[ H^{+} \right]}- \left[ H^{+} \right]$ (5)

The likelihood of CaCO_3_ precipitating or dissolving can be predicted using knowledge of the relevant dissolved ion concentrations and mineral solubility product (K_sp_):

$\mathrm{CaCO}_{3} \rightleftharpoons\mathrm{Ca}^{2+}+ \mathrm{CO}_{3}^{2-}$ $K_{\mathrm{sp}}= \left[ \mathrm{Ca}^{2+} \right]\left[ \mathrm{CO}_{3}^{2-} \right]={10}^{-8.48(calcite)}$ (6)

For the ion activities in soil solutions an Ion Activity Product (IAP) can be calculated:

$\mathrm{IAP}_{\mathrm{gypsum}}= \left[ \mathrm{Ca}^{2+} \right]\left[ \mathrm{CO}_{3}^{2-} \right]$ (7)

and the mineral saturation state may be expressed as the ratio between IAP and K_sp_ which is termed the saturation index (SI):

$SI=log\left( \frac{\mathrm{IAP}}{K_{\mathrm{sp}}} \right)$ (8)

For SI = 0, there is equilibrium between the mineral and the solution. SI < 0 reflects subsaturation (no new precipitation but potential for dissolution if mineral already present), and SI > 0 supersaturation (mineral precipitation likely).

**References**

1. Stumm W, Morgan J. Aquatic chemistry: Chemical equilibria and rates in natural waters, 3rd ed. New York: Wiley Interscience; 1996.
2. Breemen NV, Mulder J, Driscoll CT. Acidification and alkalinization of soils Plant and Soil. Vol 75, 1983. pp. 283-308.
